# MEDITERRANEAN DIET ADHERENCE PROSPECTIVELY PREDICTS NINE-YEAR CHANGES IN EPISODIC MEMORY

**DOI:** 10.1093/geroni/igad104.3109

**Published:** 2023-12-21

**Authors:** Kelly Parker, Ryan McGrath, Yeong Rhee, Jeremy Hamm

**Affiliations:** North Dakota State University, Fargo, North Dakota, United States; North Dakota State University, Fargo, North Dakota, United States; North Dakota State University, Fargo, North Dakota, United States; North Dakota State University, Fargo, North Dakota, United States

## Abstract

Mediterranean diet (MD) adherence has been shown to foster cognitive function during aging. Methods for measuring adherence to the MD typically provide or withhold points for foods consumed. Few investigations have examined how adherence to a Western adaptation of the MD may influence cognitive function over time in the United States. A modified MD that includes dairy products, whole grains, and plant-based proteins with limited consumption of sugar-sweetened beverages may be more attainable for many Americans than the traditional MD. This secondary analysis examined whether adherence to a modified MD was predictive of episodic memory and executive function over a 9-year study period. The second wave of the Midlife in the United States Study assessed general diet qualities with a food frequency questionnaire, and cognitive function was measured at both waves 2 and 3 (N = 816; age: 46 +/- 12 years; 56% female). Regression analyses were conducted to determine whether MD adherence predicted 9-year regressed change in cognitive function while controlling for age, sex, race, socioeconomic status (SES), and biomarkers of glucose homeostasis (hemoglobin a1c, insulin, and blood glucose). MD predicted changes in episodic memory (β = 0.075, p = 0.013) but not executive functioning (β = -0.028, p = 0.210). Exploratory moderator analyses suggested that MD associations with episodic memory were observed for only younger and middle-aged adults, females, White individuals, and persons with lower SES. Increased MD adherence may serve as a modifiable factor that supports cognitive function and preserved episodic memory during aging.

